# Haemodynamic effects of inhaled nitric oxide in acute myocardial infarction complicated by right heart failure under ECPELLA support: case report

**DOI:** 10.1093/ehjcr/ytad369

**Published:** 2023-08-02

**Authors:** Kosuke Fujita, Masafumi Ueno, Masakazu Yasuda, Kazuki Mizutani, Tatsuya Miyoshi, Gaku Nakazawa

**Affiliations:** Department of Cardiology, Kindai University Hospital, 377-2 Onohigashi Osakasayamashi, 589-8511 Osaka, Japan; Department of Cardiology, Kindai University Hospital, 377-2 Onohigashi Osakasayamashi, 589-8511 Osaka, Japan; Department of Cardiology, Kindai University Hospital, 377-2 Onohigashi Osakasayamashi, 589-8511 Osaka, Japan; Department of Cardiology, Kindai University Hospital, 377-2 Onohigashi Osakasayamashi, 589-8511 Osaka, Japan; Department of Cardiology, Kindai University Hospital, 377-2 Onohigashi Osakasayamashi, 589-8511 Osaka, Japan; Department of Cardiology, Kindai University Hospital, 377-2 Onohigashi Osakasayamashi, 589-8511 Osaka, Japan

**Keywords:** Inhaled nitric oxide, ECPELLA, Acute myocardial infarction, Impella, VA-ECMO, Case report

## Abstract

**Background:**

Recently, mechanical support obtained with the combination of venoarterial extracorporeal membrane oxygenation (VA-ECMO) and an Impella device, together referred to as ECPELLA, has been shown to be effective for acute myocardial infarction with cardiogenic shock. However, methods for withdrawing VA-ECMO in acute myocardial infarction cases complicated by right ventricular dysfunction are yet to be established. Here, we report the effective use of inhaled nitric oxide during the weaning of VA-ECMO from the ECPELLA management of a patient with acute myocardial infarction with cardiogenic shock.

**Case summary:**

An 81-year-old man with an acute extensive anterior wall myocardial infarction with cardiogenic shock was supported with ECPELLA to improve his haemodynamics. During ECPELLA, the Impella device could not maintain sufficient flow. Echocardiography revealed a small left ventricle and an enlarged right ventricle, indicating acute right heart failure. Inhaled nitric oxide was initiated to reduce right ventricle afterload, which decreased pulmonary artery pressure from 34/20 to 27/13 mmHg, improved right and left ventricle sizes, and stabilized the Impella support. Afterward, VA-ECMO could be withdrawn because the Impella alone was sufficient for haemodynamic support.

**Discussion:**

Inhaled nitric oxide improved right ventricle performance in a patient with severe myocardial infarction with right heart failure supported by ECPELLA. Thus, we suggest that inhaled nitric oxide facilitates the weaning of VA-ECMO from patients with refractory right ventricular dysfunction who are supported by ECPELLA.

**Learning points**

- Efficacy of inhaled nitric oxide for right ventricular dysfunction during withdrawal venoarterial extracorporeal membrane oxygenation from ECPELLA.

## Introduction

In recent years, mechanical support provided by venoarterial extracorporeal membrane oxygenation (VA-ECMO) in combination with an Impella device, together referred to as ECPELLA, has been reported to be more effective than VA-ECMO alone for acute myocardial infarction (AMI) patients with cardiogenic shock.^1^ However, no method has been established for the weaning of such patients from VA-ECMO in cases with complications of right ventricular (RV) dysfunction. Previous studies have reported inhaled nitric oxide (iNO) to improve acute haemodynamics when administered to patients with RV myocardial infarction and cardiogenic shock.^2^ Here, we reported a case of effective iNO use during VA-ECMO withdrawal from the ECPELLA management of a patient with AMI with cardiogenic shock.

## Summary figure

**Figure:**
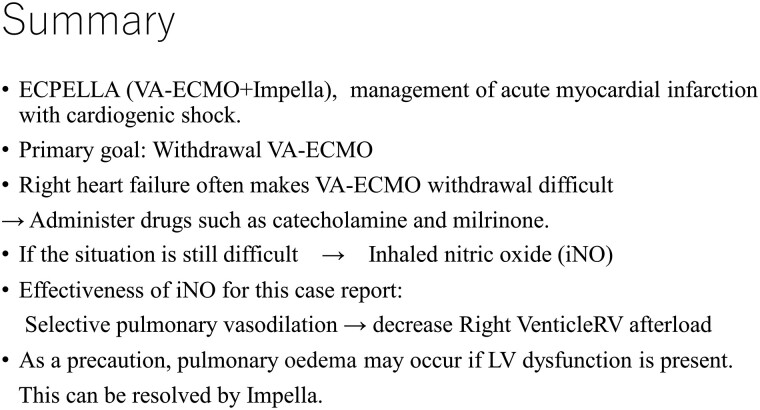


## Case presentation

**Figure 1:**
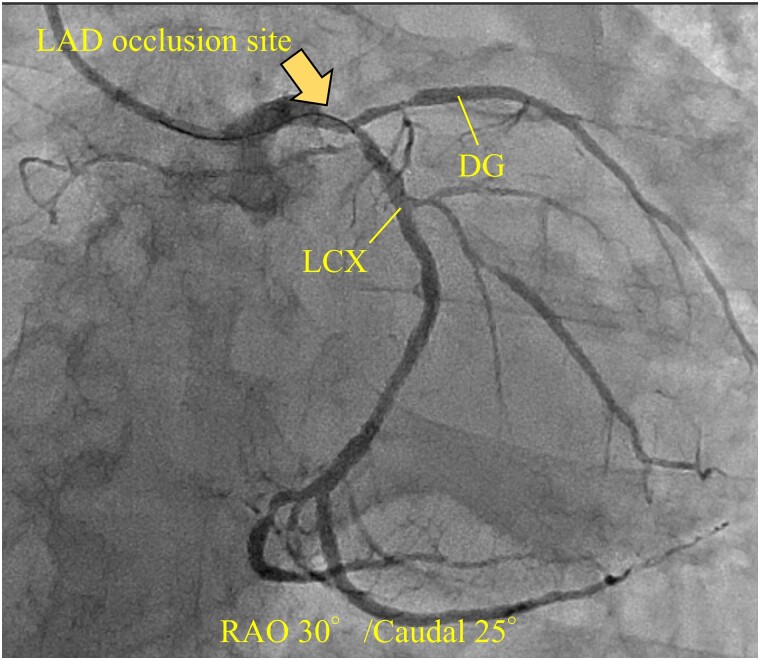
Coronary angiography of left coronary artery (right anterior oblique [RAO] 30°/Caudal 25°). It shows that complete occlusion of the proximal left anterior descending (LAD) artery and significant stenosis of the left main coronary artery (LMCA), proximal left circumflex (LCX) artery and proximal diagonal branch (DG). LAD is completely occluded from the site indicated by the arrow.

**Figure 2:**
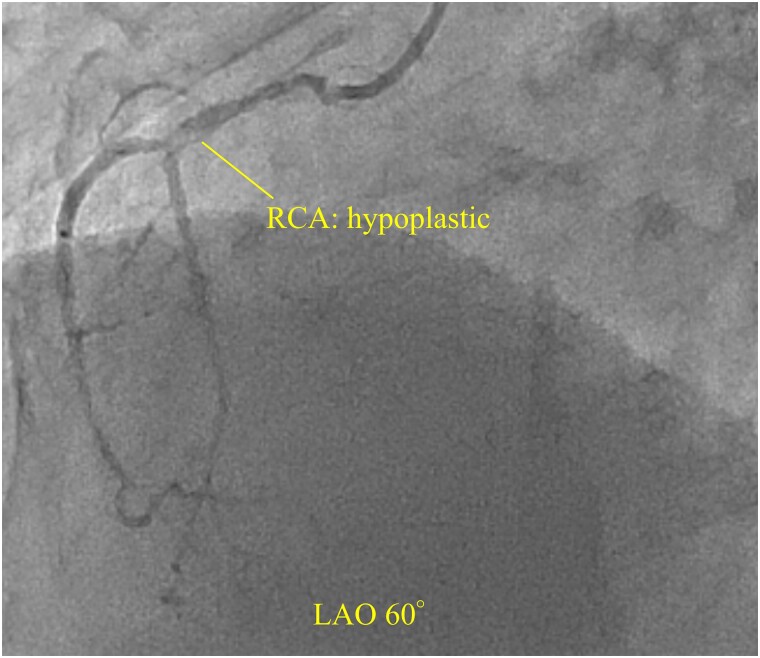
Coronary angiography of right coronary artery (left anterior oblique 60°). It shows that the right coronary artery was hypoplastic. LAO, left anterior oblique; RCA, right coronary artery.

**Figure 3:**
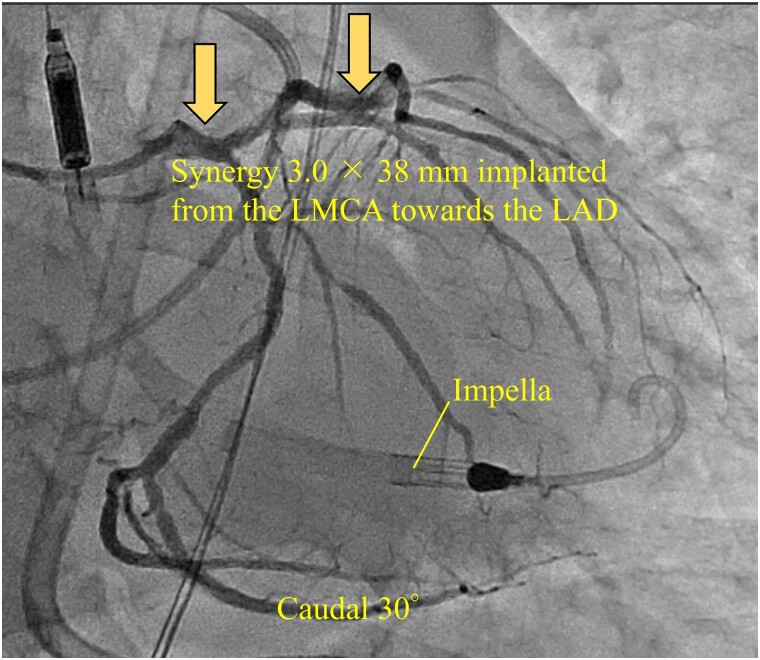
It shows post-percutaneous coronary intervention findings (Caudal 30°). The percutaneous coronary intervention was completed with a drug-eluting stent (everolimus-eluting stent, Synergy 3.0 × 38 mm) implantation across the left circumflex bifurcation from the left main coronary artery towards the left anterior descending. The two arrows indicate the proximal and distal of the stent. LAD, left anterior descending; LCX, left circumflex; LMCA, left main coronary artery.

An 81-year-old man with a history of hypertension and dyslipidaemia was admitted to the hospital with acute chest pain. At presentation, the blood pressure and heart rate were 116/70 mmHg and 64 b.p.m. Physical examination revealed no heart murmur or pulmonary rales. Electrocardiography showed an elevated ST segment in leads V_1_–V_4_, and transthoracic echocardiography depicted severe hypokinesis of the anterior wall and septum. Coronary angiography showed complete occlusion of the proximal left anterior descending (LAD) artery and significant stenosis of the left main coronary artery (LMCA) and proximal left circumflex (LCX) artery (*Figure 1*). The right coronary artery was hypoplastic (*Figure 2*). Percutaneous coronary intervention (PCI) was initiated considering the LMCA–LAD lesion as the responsible lesion. After performing the kissing balloon technique in both the LAD and LCX, successful PCI with implantation of a drug-eluting stent (everolimus-eluting stent, Synergy 3.0 × 38 mm) across the LCX bifurcation from the LMCA to the LAD (*Figure 3*). During PCI, the patient went into shock and required escalating doses of inotropes/vasopressors and an intra-aortic balloon pump (IABP). These procedures, however, did not improve the patient’s haemodynamic condition, and VA-ECMO insertion was decided. After completing PCI, IABP was upgraded to an Impella device to unload the left ventricle (LV) and decrease its oxygen consumption. Creatine kinase (CK; 13 609 kat/L) and CK-MB (1512 kat/L) were at their peak levels for 9 h from onset.

During ECPELLA, the Impella device could not maintain sufficient flow and Impella suction occurred on Day 3. Transthoracic echocardiogram revealed a small LV and an enlarged RV, suggesting acute RV failure (see Supplementary material online, *Video S1*). We introduced 0.5 µg/kg/min of PDE3 inhibitor in addition to dobutamine and noradrenalin to manage RV failure. However, we were unable to achieve sufficient improvement in RV failure.

**Figure 4:**
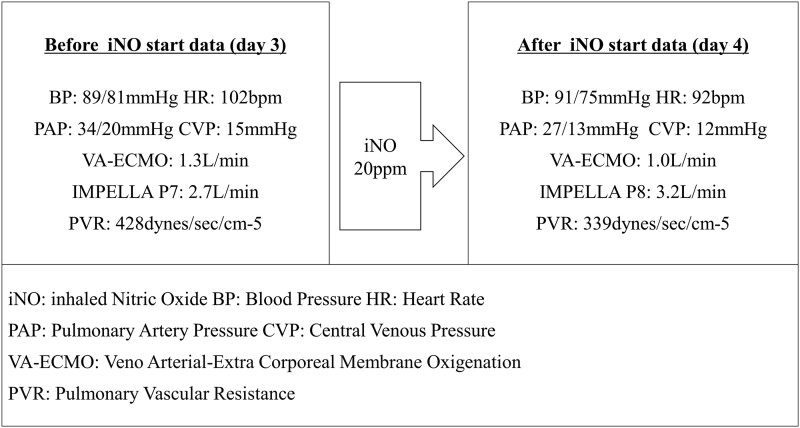
During ECPELLA, blood pressure, heart rate, pulmonary artery pressure, central venous pressure, venoarterial extracorporeal membrane oxygenation flow, Impella flow, and pulmonary vascular resistance before (on Day 3) and after inhaled nitric oxide start (on Day 4).

Inhaled nitric oxide at a maximum dose of 20 ppm was initiated to reduce RV afterload on Day 3. A decrease was observed in pulmonary artery pressure and vascular resistance from 34/20 to 27/13 mmHg and from 428 to 339 dynes/s/cm^5^, respectively. Furthermore, an improvement in the RV and LV sizes was noted (see Supplementary material online, *Video S2*) and the stabilization of the Impella support (*Figure 4*). Afterward, VA-ECMO could be withdrawn as the Impella device alone was sufficient for haemodynamic support. We successfully withdrew VA-ECMO on Day 4. Following withdrawal, the Impella device exhibited a cardiac output of 3.5 L in the P8 setting and the pulmonary artery pulsatility index was 1.2. The tendency towards hypotension, despite adequate maintenance of the intravascular volume and cardiac output, led to the conclusion that the patient was experiencing septic shock. Laboratory tests showed an increased white blood cell count (10 820/µL) and C-reactive protein level (22.67 mg/dL).

Intravenous vasopressin and antimicrobials were administered for alleviating septic shock. Gradually, the inflammatory marker levels decreased and the doses of vasopressin and catecholamines were reduced; however, alarms indicating increased purge pressure in the Impella device appeared on Day 7. The Impella device was at risk of stopping abruptly. At this stage, it was decided that the patient could not be weaned off the Impella device and the device was upgraded from IMPELLACP to IMPELLA5.0. The patient was changed the Impella to IABP on Day 12 and then successfully weaned off IABP on Day 15. Furthermore, the patient was weaned off the ventilator on Day 18, starting weaning rehabilitation. Finally, inotrope administration was eventually discontinued, and the patient was transferred to a rehabilitation hospital on Day 79. The patient was discharged home after about 1-month rehabilitation programme and is currently being regularly followed in the cardiological outpatient clinic: his condition appears stable.

## Discussion

The effectiveness of ECPELLA in post-AMI patients with cardiogenic shock has been demonstrated in previous studies.^1^ The potential of combining Impella and VA-ECMO to effectively facilitate LV unloading has been described in five cases summarized in a previously published case report.^3^ Although our patient exhibited a significant degree of myocardial damage, LV unloading using an Impella device facilitated the management of heart failure in the acute and chronic phases. Conversely, ECPELLA, especially with VA-ECMO, despite appropriate anticoagulation precautions, is often fatal because of bleeding, thrombosis, infection, and other complications.^4^ Therefore, early weaning from VA-ECMO should be encouraged. During ECPELLA support in our patient, medication, including milrinone, did not improve RV failure, thereby making VA-ECMO withdrawal challenging. In the present case, iNO was highly effective in improving RV dysfunction. There have been case reports highlighting a similar efficacy of iNO in ECPELLA for fulminant myocarditis and cardiogenic shock following cardiac surgery.^5,6^ Our case presents a similar situation to these two case reports.

The common situation is the management of ECPELLA in both heart failures and the difficulty in VA-ECMO withdrawal due to RV dysfunction.

In addition, initiating or increasing iNO decreases pulmonary artery pressure and vascular resistance, improves right heart failure, stabilizes support with Impella, and weans the patient off VA-ECMO. In both case reports, the patient had prolonged right heart dysfunction due to the underlying disease, requiring pulmonary vasodilators at the time of iNO withdrawal. In our case, although the patient did not have right coronary artery occlusion, he exhibited extensive anterior myocardial infarction due to LAD occlusion accompanied by RV dysfunction. It has been suggested that RV myocardial infarction can occur because of left coronary artery occlusion. In cases of left coronary artery predominance, LCX ischaemia can induce RV myocardial infarction.^7,8^ It has also been reported that acute LV failure with cardiogenic shock can cause RV failure in a study of pigs. When microspheres were injected into the LMCA to induce cardiogenic shock, the LV went into profound failure, but the RV performance was also severely impaired, as demonstrated by ventricle-arterial decoupling in both ventricles.^9^

A study involving a porcine model showed that iNO in addition to assisted circulation using an Impella device improved RV failure and confirmed that iNO successfully decreased RV afterload, normalized RV filling pressure over time, and shifted the RV strain towards a normal configuration.^10^

Inhaled nitric oxide selectively vasodilates pulmonary vessels via cyclic guanosine monophosphate production in pulmonary smooth muscle cells, and its ability to decrease pulmonary artery pressure and vascular resistance and improve gas exchange has led to its use in the management of RV failure. Nitric oxide is scavenged by haemoglobin on diffusing into the blood and is thereby rapidly inactivated; the vasodilatory effect of iNO is limited to the lung, thus preventing systemic hypotension.^11,12^ Inhaled nitric oxide reduced RV afterload, which enabled increased blood flow from the RV to the LV to normalize the LV volume and increase the flow of the Impella device. Notes, in patients with LV dysfunction, iNO may induce pulmonary oedema by decreasing pulmonary vascular resistance and increasing pulmonary arterial wedge pressure.^5^

Our case also had LV dysfunction, but pulmonary oedema was prevented by LV functional support with Impella. Hence, iNO is not indicated for all cases of right heart failure but should be limited to patients with preserved left heart function or mechanical assistance.

## Conclusion

Inhaled nitric oxide improved RV function in severe myocardial infarction patients complicated by right heart failure who were provided with ECPELLA support. Hence, we suggest that iNO is effective for VA-ECMO withdrawal in patients with refractory RV dysfunction who are supported with ECPELLA.

The findings of this case should be further corroborated with those of other similar cases in the future.

## Supplementary Material

ytad369_Supplementary_Data

## Data Availability

All relevant data supporting the conclusions of this article are included within the article.

## Lead author biography

Dr Fujita graduated from Kindai University Hospital in 2010. He is working at Kindai university Hospital, Department of Cardiology. His speciality is ischemic heart disease and structural heart disease.

## Supplementary material

**Consent:** We obtained written and signed consent in accordance with COPE guidelines.

**Funding:** This study was not funded.
