# Supplementary material for: Haemodynamic effects of inhaled nitric oxide in acute myocardial infarction complicated by right heart failure under ECPELLA support: case report
Source: Eur Heart J Case Rep. 2023 Aug 2;7(8):ytad369. doi: 10.1093/ehjcr/ytad369 (PMC10422691; doi:10.1093/ehjcr/ytad369)
Supplement: ytad369_Supplementary_Data [file ytad369_supplementary_data.zip › author signature.pdf]

Request for additional authors above article limits

This form should be used by the corresponding author if they wish to request that additional authors are required above the limits outlined by the journal. Please note that submission of this form does not guarantee agreement by the editors of EHJ-Case Reports to allow additional authors to be included.

All authors should be listed and their contributions defined. All authors are required to meet the 'authorship criteria' to be considered appropriate for inclusion. A reason for requesting more authors than than the authorship limit is required and should be selected from the dropdown list, please note the requirement to provide further details for these selections

**Article Title**	Hemodynamic effects of inhaled nitric oxide in acute myocardial infarction complicated by right heart failure		
**Article reference (if available)**	[1] Pappalardo F, Schulte C, Pieri M, Schrage B, Contri R, Soeffker G, et al. Concomitant implantation of		
**Article Type**	Case report	Author Limit	4
**Corresponding Author name**	Kosuke Fujita		
**Full author list (in the order you would like them to appear)**	Kosuke Fujita, Masafumi Ueno, Masakazu Yasuda, Kazuki Mizutani, Tatsuya Miyoshi, Gaku Nakazawa		
**Reason for requesting additional authors**	patient care required essential involvement of multiple sub-specialties from within cardiology		
**Additional details**			

Order	Author name	Author contribution				Meets authorship criteria	Notes	Author signature
		Involvement with patient care	Manuscript preparation	Final approval	Accountability			
1	Kosuke Fujita	Patient Care - Undertook	Drafting manuscript	Final approval of the manuscript	Agreement to be accountable for all aspects of the work	YES		
2	Masafumi Ueno	Patient Care - Supervised	Critical revision of the manuscript	Final approval of the manuscript	Agreement to be accountable for all aspects of the work	YES		
3	Masakazu Yasuda	Patient Care - Undertook	Critical revision of the manuscript	Final approval of the manuscript	Agreement to be accountable for all aspects of the work	YES		
4	Kazuki Mizutani	Patient Care - Supervised	Critical revision of the manuscript	Final approval of the manuscript	Agreement to be accountable for all aspects of the work	YES		
5	Tatsuya Miyoshi	Patient Care - Supervised	Critical revision of the manuscript	Final approval of the manuscript	Agreement to be accountable for all aspects of the work	YES		
6	Gaku Nakazawa	Patient Care - Supervised	Critical revision of the manuscript	Final approval of the manuscript	Agreement to be accountable for all aspects of the work	YES		
7								
8								
9								
10								
